# Haemodynamics and oxygenation improvement induced by high frequency percussive ventilation in a patient with hypoxia following cardiac surgery: a case report

**DOI:** 10.1186/1752-1947-4-339

**Published:** 2010-10-25

**Authors:** Alessandro Forti, Valeria Salandin, Paolo Zanatta, Bruno Persi, Carlo Sorbara

**Affiliations:** 1 Anesthesia and Intensive Care Department, Treviso Regional Hospital, Piazza Ospedale No 1, 31100 Treviso, Italy

## Abstract

**Introduction:**

High frequency percussive ventilation is a ventilatory technique that delivers small bursts of high flow respiratory gas into the lungs at high rates. It is classified as a pneumatically powered, pressure-regulated, time-cycled, high-frequency flow interrupter modality of ventilation. High frequency percussive ventilation improves the arterial partial pressure of oxygen with the same positive end expiratory pressure and fractional inspiratory oxygen level as conventional ventilation using a minor mean airway pressure in an open circuit. It reduces the barotraumatic events in a hypoxic patient who has low lung-compliance. To the best of our knowledge, there have been no papers published about this ventilation modality in patients with severe hypoxaemia after cardiac surgery.

**Case presentation:**

A 75-year-old Caucasian man with an ejection fraction of 27 percent, developed a lung infection with severe hypoxaemia [partial pressure of oxygen/fractional inspiratory oxygen of 90] ten days after cardiac surgery. Conventional ventilation did not improve the gas exchange. He was treated with high frequency percussive ventilation for 12 hours with a low conventional respiratory rate (five per minute). His cardiac output and systemic and pulmonary pressures were monitored.

Compared to conventional ventilation, high frequency percussive ventilation gives an improvement of the partial pressure of oxygen from 90 to 190 mmHg with the same fractional inspiratory oxygen and positive end expiratory pressure level. His right ventricular stroke work index was lowered from 19 to seven g-m/m^2^/beat; his pulmonary vascular resistance index from 267 to 190 dynes•seconds/cm^5^/m^2^; left ventricular stroke work index from 28 to 16 gm-m/m^2^/beat; and his pulmonary arterial wedge pressure was lowered from 32 to 24 mmHg with a lower mean airway pressure compared to conventional ventilation. His cardiac index (2.7 L/min/m^2^) and ejection fraction (27 percent) did not change.

**Conclusion:**

Although the high frequency percussive ventilation was started ten days after the conventional ventilation, it still improved the gas exchange. The reduction of right ventricular stroke work index, left ventricular stroke work index, pulmonary vascular resistance index and pulmonary arterial wedge pressure is directly related to the lower respiratory mean airway pressure and the consequent afterload reduction.

## Introduction

Lung injury is a well-recognized complication after operations for cardiac surgery [1]. Cardiopulmonary bypass leads to the activation of complement, neutrophils, monocytes, macrophages, platelets and endothelial cells with secretion of cytokines, proteases, arachidonic acid metabolites and oxygen-free radicals. Leukocyte adhesion to microvascular endothelium, leukocyte extravasation and tissue damage can be seen in the final stages [2]. Major thoracic and abdominal surgery significantly reduces the respiratory reserve. Postoperative pulmonary complications, such as atelectasis and pneumonia, seem to be related to the disruption of the normal activity of the respiratory muscles. The disruption begins with the induction of anaesthesia and may continue into the post-operative period. Anaesthetics and drugs used in the peri-operative period affect the central regulation of breathing and change the neural drive to the respiratory muscles and, in particular, to the diaphragm [3]. On the first postoperative day after a sternotomy, the observed decrease in forced vital capacity (FVC) is reported to be around 70% of the preoperative value. Ten days after surgery, when most patients can be discharged from the hospital, the FVC has increased but still remains at 30% lower than the preoperative value [4]. It has been reported that high frequency percussive ventilation (HFPV) improves gas exchange where normal ventilation and lung recruitment therapy have failed.

HFPV VDR4 (Percussionaire Bird Technologies, ID, USA) is a ventilatory technique that delivers small bursts of high flow respiratory gas into the lung at high rates. It is classified as a pneumatically-powered, pressure-regulated, time-cycled, high-frequency flow interrupter modality of ventilation. The core of this system is the phasitron, which acts as a piston mechanism. The piston switches a high-pressure gas supply at a frequency rate of 200-1200 bpm to a low pressure rate, with high gas flow velocity.

During inspiration, lung volumes are progressively increased in a controlled, stepwise fashion by repeatedly fading subtidal volume deliveries until an oscillatory plateau is reached and maintained [5]. At the end of inspiration, the lung is allowed to empty passively until a preset expiratory base-line is reached.

It has been noted that gas exchange is as good as, if not better than, conventional ventilation (CV) at lower airway pressures. As described by Krishnan and Brower [6], there are six mechanisms that may contribute to gas exchange during all forms of high frequency ventilation: (1) direct bulk flow - the flow of inspired air in proximal alveoli leading to gas exchange by traditional methods (as with CV); (2) longitudinal dispersion is secondary to mixing from turbulent and swirling flow patterns; (3) variable flow is directed between adjacent lung regions with differences in compliance and resistance; (4) asymmetric velocity profiles - the laminar flow pattern in which gas in the centre of the airway advances inward and gas outside the centre flows in a retrograde way; (5) cardiogenic mixing - mechanical agitation from the normal heart beat, especially in peripheral lung units; and (6) molecular diffusion - the mixing of air in the smallest lung units near the alveolo-capillary membrane. HFPV is designed to be used in conjunction with mechanical ventilation or as a stand-alone treatment. This is the first case report of this ventilation modality in a patient with severe hypoxaemia after cardiac surgery.

## Case presentation

A 75-year-old Caucasian man developed a lung infection with severe hypoxaemia [arterial partial pressure of oxygen (PaO_2_)/fractional inspiratory oxygen (FiO_2_) of 90] ten days after aortic valve replacement due to a severe aortic stenosis. He weighed 80 Kg and had a body surface area of 1.98 m^2^, an ejection fraction (EF) of 27 percent and a history of post ischaemic dilated cardiomiopathy, severe aortic stenosis with a mean gradient of 63 mmHg, hypertension and insulin dependent diabetes mellitus - there had been no alcohol or tobacco use in the last ten years. We do not have the data for the preoperative gas exchange data or the haemodynamic data; we only have the preoperatory lung function test, which shows a moderate obstructive disease.

The patient was operated via a median sternotomy. The aortic valve was replaced with a biological Hancock 21 mm valve. The weaning from the extracorporeal circulation (ECC) was performed with an intraortic balloon pump with a high dose of inotropic drugs (norephinefrine 0.25 μg/kg/min and levosimendan 0.2 μg/kg/min). The time taken for the intervention was 260 min globally, including 55 min of aortic clamp and 125 min of ECC. The total fluid balance at the end of the operation was +1000 mL.

On the second post-operative day PaO_2_/FiO_2 _slowly decreased to 90 on the tenth day. We performed CV in increased pulmonary residual volume modality (Dräger Evita XL) with recruitment manoeuvre, high positive end expiratory pressure (PEEP) level (14 cmH_2_O), low tidal volume (6-8 mL/Kg), peak inspiratory pressure (PIP) of 38 cmH_2_O, mean airway pressure (MArP) of 24 cm H_2_O without any significative increase of respiratory parameter. Cardiac output, systemic and pulmonary pressures, were monitored. The patient was ventilated for four days in a pressure regulated volume controlled modality.

On days 3 and 4 we started a recruitment manoeuvre in the pressure-controlled mode at an inspiratory plateau pressure of 45 cm of water, a PEEP of five cm of water, a respiratory rate of ten breaths per minute and a 1:1 ratio of inspiration to expiration for two minutes. After the recruitment manoeuvre, PEEP at a level of 14 cm of water was applied. The PEEP level of 14 cm of water reflects the upper inflection point on the deflation limb of the pressure/volume curve and it can be used to prevent alveolar re-collapse and instability; after that we switch into pressure support ventilation but with an unsatisfactory gas exchange.

On day six we restarted with pressure regulated volume-controlled modality for two days. On days eight to ten we began bi-level positive airway pressure ventilation but it did not have an acceptable effect.

On day 11 HFPV was started with: a 650/min percussive rate; a convective rate of 5/min; 14 cm H_2_O PEEP; 46 cm H_2_O PIP; 2.0 sec inspiratory time; 10.8 sec expiratory time; 16 cm H_2_O MArP; 1:7.0 inspiratory-expiratory (I:E) rate of conventional ventilation; and 1:1.0 I:E rate of the micro percussive burst. After only two hours of HFPV we noted an improvement of PaO_2 _from 90 to 190 mmHg with the same FiO_2 _and PEEP level of conventional ventilation. His right ventricular stroke work index **(**RVSWI) was lowered from 19 to 7 g-m/m^2^/beat, pulmonary vascular resistance index (PVRI) from 267 to 190 dynes•sec/cm^5^/m^2^, left ventricular stroke work index (LVSWI) from 28 to 16 g-m/m^2^/beat, pulmonary artery wedge pressure (PAWP) from 32 to 24 mmHg with a lower MArP than with conventional ventilation. The cardiac index (2.7 L/min/m^2^) and ejection fraction (EF) of 27% did not change. Diuresis was always maintained between 1-1.5 mL/kg/hour. After 12 hours of HFPV the tidal volume increased from 600 to 750 mL, MArP was lowered from 24 to 20 cmH_2_O, FiO_2 _from 1% to 0.6% and PIP from 38 to 34 cmH_2_O, with conventional ventilation. After 12 hours of HFPV we reconnected the patient to the conventional ventilation and ten hours later he was successfully extubated. Two days later he was admitted to the subintesive care unit.

We noted that HFPV (Percussionaire Bird Technologies, ID, US) improved oxygenation and it had an effect after only two hours of therapy. Reper *et al. *[7] show the same results in a burn patient. Another study showed better secretion clearance and outcome when using HFPV during thoracotomy [8]. Both these mechanisms improve gas exchange. Swan Ganz catheter with a Vigilance^© ^(Edward, CA, US) monitor was used to measure cardiac output and systemic and pulmonary pressures. We detected the haemodynamic and respiratory parameter after two, six and 12 hours of unconventional ventilation therapy (Tables 1, 2 and 3).

**Table 1:** Oxygenation and haemodynamic improvement

	CV	HFPV after 2 hours	HFPV after 6 hours	HFPV after 12 hours	CV after HFPV
Ph	7.51	7.48	7.44	7.4	7.41
pCO_2_(mmHg)	49	47	46	43	45
PaO_2 _(mmHg)	89	190	189	145	140
FiO_2_(%)	1	1	0.8	0,6	0.6
RVSWI (g-m/m^2^/beat)	19	14	7	7	10
LVSWI (g-m/m^2^/beat)	28	17	16	17	21
PVRI (dynes•sec/cm^5^/m^2)^	267	190	192	195	240
PAWP (mmHg)	32	24	25	25	30
CI	2,7	2,7	2,7	2,6	2,5

PaO_2 _rose from 90 to 190 mmHg with the same fractional inspiratory oxygen (FiO_2_) and positive end expiratory pressure level of conventional ventilation. Right ventricular stroke work index (RVSWI) lowered from 19 to 7

g-m/m^2^/beat, pulmonary vascular resistance index (PVRI) from 267 to 190 dynes•sec/cm^5^/m^2^, left ventricular stroke work index (LVSWI) from 28 to 16 g-m/m^2^/beat, pulmonary artery wedge pressure (PAWP) from 32 to 24 mmHg with a lower mean airway pressure than conventional ventilation. Cardiac index (2.7 L/min/m^2^) and ejection fraction (EF) of 27% did not change.

CV, conventional ventilation; HPFV, high frequency percussive ventilation.

**Table 2:** Ventilator setting pre- and post-high frequency percussive ventilation (HFPV)

	VGRP pre HFPV	VGRP after HFPV
TV (mL)	600	750
MArP (cm H_2_O)	24	20
Respiratory	12	12
rate (rate/min)		
FiO_2_(%)	1	0.6
PEEP (cm H_2_O)	14	14
PIP (cm H_2_O)	38	34
Inspiratory-expiratory rate	1:1.5	1:1.5

After 12 hours of HFPV, tidal volume (TV) increased from 600 to 750 mL, mean airway pressure (MArP) lowered from 18 to 16 cmH_2_O, fractional inspiratory oxygen (FiO_2_) from 1 to 0.6%, peak inspiratory pressure (PIP) from 38 to 36 cmH_2_O.

**Table 3:** High frequency percussive ventilation (HFPV) setting

	HFPV post 2 hours	HFPV post 6 hours	HFPV post 12 hours
Percussive rate(rate/min)	650	650	650
Convective rate(rate/min)	5	5	5
PEEP (cm H_2_O)	14	14	14
PIP (cm H_2_O)	46	46	43
MArP (cm H_2_O)	16	16	13
IT (sec)	2,0	1,9	2,1
ET (sec)	10.8	10.9	10.7
I:E	1:7.0	1:6.9	1:7.1
i:e	1:1	1:1	1:1
FiO_2_(%)	1	0.8	0.6

The high peak inspiratory pressure (PIP) level does not indicate a very high-pressure level, because the sample point is on the patient, directly connected with the endotracheal tube. PIPs at the carina are approximately one-third the level set on the HFPV. In the conventional ventilators the sampling point is inside the ventilator, 1.80 m away from the patient, the mean airway pressure (MArP) measure depends on the dissipated energy through the ventilator tubes.

PEEP, positive end expiratory pressure.

**Figure 1:**
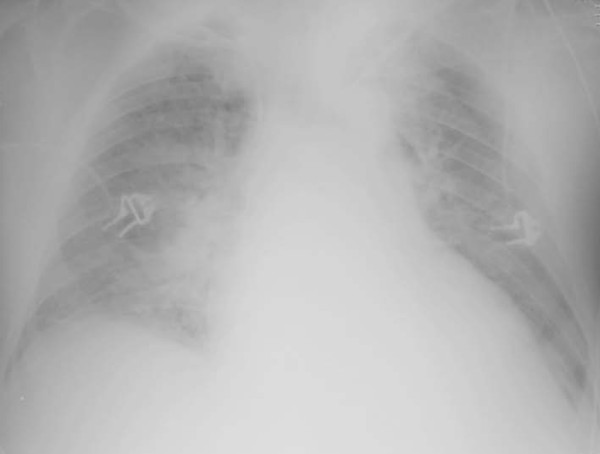
**The patient before high frequency percussive ventilation**.

**Figure 2:**
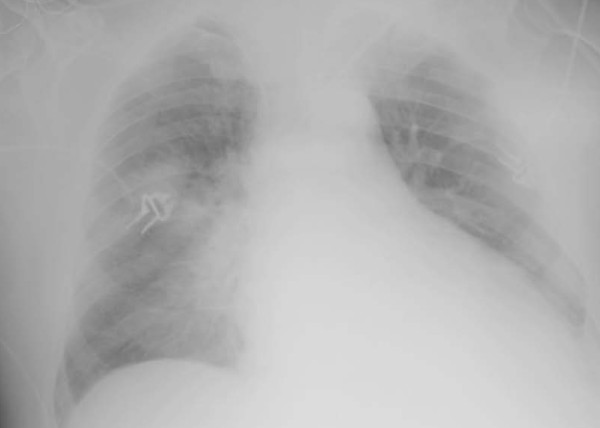
**The patient after high frequency percussive ventilation treatment**.

**Figure 3:**
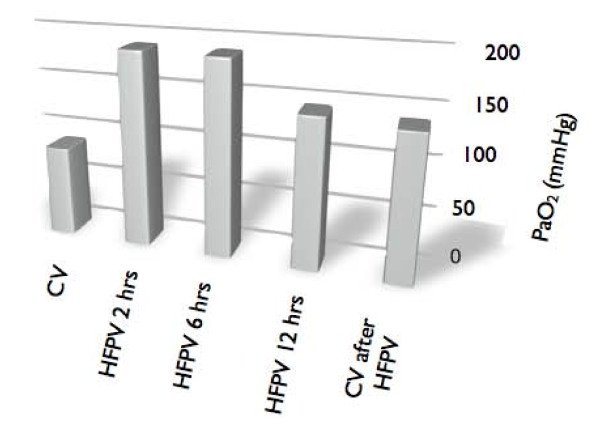
**Arterial partial pressure of oxygen increasing after high frequency percussive ventilation treatment**.

The chest X-ray (Figures 1 and 2) shows an improvement on the right lung compared to the preceding day and after only 12 hours of HFPV. Figure 3 shows the PaO_2 _increasing after HFPV.

Compared to conventional ventilation, HFPV gave an improved PaO_2 _of from 90 to 190 mmHg after only two hours and with the same PEEP and FiO_2 _level as conventional ventilation. Velmahos *et al*. [9] reported a series of 32 adult medical and surgical intensive-care unit patients with acute lung distress syndrome who were failing with conventional ventilation (CV). In our case, the mean PaO_2_/FiO_2 _on CV was 111, which was improved to 163 after one hour by converting to HFPV and 193 at 48 hours. PIP decreased from 42.4 cm H_2_O on CV to 33.2 cm H_2_O after one hour of HFPV and 32.5 at 48 hours, but the MArP increased from 21 cm H_2_O on CV to 27 cm H_2_O on HFPV. There was no change in haemodynamic variables. The tidal volume increased as a result of the increasing lung compliance which had been improved by the HFPV.

We found that there was a decrease in the RVSWI, LVSWI, PVRI and PAWP due to a reduction of MArP compared to the CV resulting in a lower afterload.

## Conclusion

This case report shows the improvement in oxygenation and ventilation in a cardiac surgery patient. To the best of our knowledge, there has been no previous published report on HFPV in cardiac surgery intensive care. Lung injury is a frequent postoperative complication in such patients. HFPV is a safe ventilatory modality that improves gas exchange when CV does not work. In patients with an acute respiratory distress syndrome the intrathoracic pressure is greater than for a normal ventilated lung.

An augmented intrathoracic pressure increases the afterload and reduces the stroke volume of the right ventricle with an increased systolic pulmonary pressure due to an increase in the pulmonary vessels resistance. It is important to reduce the mean airway pressure and decrease the interference to the cardiac cycle.

With HFPV the mean airway pressure is lower than with conventional ventilation and so it may, therefore, improve the right ventricle function. More studies are required in order to confirm this data.

## Abbreviations

CV: conventional ventilation; EEC: extracorporeal circulation; EF: Ejection fraction; FiO_2_: fractional inspiratory oxygen; HFPV: high frequency percussive ventilation; I:E: inspiratory-expiratory rate; LVSWI: left ventricular stroke work index; MArP: mean airway pressure; PaO_2_: arterial partial pressure of oxygen; PAWP: pulmonary artery wedge pressure; PEEP: positive end expiratory pressure; PIP: peak inspiratory pressure; PVRI: pulmonary vascular resistance index; RVSWI: right ventricular stroke work index.

## Competing interests

The authors declare that they have no competing interests.

## Consent

Written informed consent was obtained from the patient for publication of this case report and any accompanying images. A copy of the written consent is available for review by the Editor-in-Chief of this journal.

## Authors' contributions

AF conceived the work, carried out the study, collected and analyzed the data and wrote the paper. PZ, VS and BP analyzed the data and helped to write the paper. CS analysed the data. All authors read and approved the final manuscript.
